# Differences in Dietary Habits, Physical Exercise, and Quality of Life between Patients with Obesity and Overweight

**DOI:** 10.3390/healthcare9070916

**Published:** 2021-07-20

**Authors:** Carmen Herrera-Espiñeira, Ana María de Pascual y Medina, Manuel López-Morales, Paloma Díaz Jiménez, Antonia Rodríguez Ruiz, Manuela Expósito-Ruiz

**Affiliations:** 1 National Network of Research in Health Departments and Chronic Diseases (REDISSEC), 18012 Granada, Spain; 2 Instituto de Investigación Biosanitaria ibs.GRANADA, 18012 Granada, Spain; malomorales@ugr.es; 3 Faculty of Health Sciences, University of Granada, 18012 Granada, Spain; 4 GREISSEC Spanish Research Group on Care in Chronic Diseases Health Services (INVESTEN), 38109 Las Palmas de Gran Canaria, Spain; anamaria.pascualmedina@sescs.es; 5 Evaluation and Planning Service of the Canary Health Service (SESCS), 38109 Las Palmas de Gran Canaria, Spain; 6 Granada-Metropolitan Health District, 18012 Granada, Spain; 7 Foundation for Biohealth Research of Eastern Andalusia (FIBAO), 18012 Granada, Spain; paloma.arteterapia@gmail.com (P.D.J.); mexpositoruiz@ugr.es (M.E.-R.); 8 Department of Internal Medicine, Motril Hospital, 18600 Motril, Spain; antoniarruiz@hotmail.com

**Keywords:** obesity, overweight, quality of life, exercise, food habits, patients, hospitalization

## Abstract

Background: Overweight and obesity differ in their repercussions on the health and health-related quality of life (HRQoL) of patients. The objective of this study was to compare physical activity levels and dietary habits before admission and HRQoL at discharge between patients with obesity and overweight. Methods: A cross-sectional study was undertaken among participants in a clinical trial on education for healthy eating and physical activity, enrolling non-diabetic patients admitted to Internal Medicine Departments. These were classified by body mass index (BMI) as having overweight (25–29.9 Kg/m^2^) or obesity (≥30 kg/m^2^). Data were gathered on sociodemographic characteristics, clinical variables (medication for anxiety/depression, Charlson Comorbidity Index, length of hospital stay), physical exercise and diet (International Physical Activity and Pardo Questionnaires), and HRQoL (EQ-5D-5L). The study included 98 patients with overweight (58.2% males) and 177 with obesity (52% males). Results: In comparison to patients with obesity, those with overweight obtained better results for regular physical exercise (*p* = 0.007), healthy diet (*p* = 0.004), and “emotional eating” (*p* = 0.017). No between-group difference was found in HqoL scores. Conclusion: Patients with overweight and obesity differ in healthy dietary and physical exercise behaviors. Greater efforts are warranted to prevent an increase in the BMI of patients, paying special attention to their state of mind.

## 1. Introduction

Overweight is defined by the World Health Organization (WHO) as a body mass index (BMI) of 25–29.9 kg/m^2^ and obesity as a BMI ≥ 30 kg/m^2^; the BMI is described as a useful but approximate measure of overweight/obesity, given that it may not correspond to the same degree of fatness in different individuals [1]. Based on this classification, it was reported in 2017 that 37.1% of adults (≥18 yrs.) in Spain were with overweight and 17.4% with obesity [2]. Obesity and overweight have mainly been attributed to an energy imbalance between the consumption and expenditure of calories, which has been exacerbated worldwide by a greater intake of foods rich in fats and sugars and a reduction in physical activity [1]. 

The risk of cardiovascular diseases, diabetes, musculoskeletal disorders, and some cancers is increased in patients with an elevated BMI [1]. This relationship between health-related quality of life (HRQoL) and BMI appears to be bidirectional, being stronger for physical than for psychological dimensions [3]. However, a study of an Asian population found that this relationship was not linear, given that a worse HRQoL has been related to both underweight and obesity, with differences between the sexes [4]. In comparison to people with overweight, it has been found that those with obesity are more prone to pain [5]. A bidirectional risk has been described between obesity and depression, which is higher in females, but not between overweight and depression [6]. 

In Spain, the sedentary habits of young people have continued to rise, with an increase in hours watching television and a reduction in physical exercise, especially among females [7]. The same trend has been observed among Spaniards aged 65 to 69 years, who do not follow WHO recommendations on physical activity [8]. The benefits of physical activity, especially aerobic exercise, have also been related to mental health [9] and positive effects on neurocognitive function [10]. Interventions to achieve weight loss focus on physical activity and/or dietary habits, and those that combine both aspects have proven the most effective [11].

Given the repercussions of obesity or overweight on the health and HRQoL of individuals, the objective of this study was to determine differences in habits related to diet and physical activity between patients with overweight and those with obesity before admission to an Internal Medicine Department and differences in their HRQoL at hospital discharge. 

## 2. Material and Methods

This cross-sectional study was undertaken among patients participating in a multi-center clinical trial on education in healthy dietary habits and physical exercise.

### 2.1. Study Population

The study included patients admitted to the Internal Medicine Department of four hospitals in Southern Spain (Virgen de las Nieves University Hospital, Granada; Baza Regional Hospital; Motril Regional Hospital; and University Hospital of Ceuta) between February 2018 and February 2020. Inclusion criteria were age ≥ 18 years, BMI ≥ 25 kg/m^2^ and absence of diabetes. People with diabetes were specifically excluded to avoid interference with or influence from the multidisciplinary plan for this disease implemented by the regional health ministry [12]. Exclusion criteria were: cognitive or physical problems hampering questionnaire completion (despite caregiver assistance); participation in a weight-loss diet plan under a nutritionist/endocrinologist; and the receipt of major surgery during the hospital stay. The final study sample comprised 275 patients. 

### 2.2. Evaluation Protocol and Variables Considered 

Patients meeting study eligibility criteria were enrolled at hospital admission, when a specifically trained nurse at each center recorded their sex and age, measured their weight and height (barefoot and in pajamas), gathered data from each patient on schooling level (no schooling or primary, secondary, or higher education), employment status (unemployed, actively employed, student, retired, or with permanent disability), cohabitation (living alone or accompanied), and pre-admission medication for anxiety or depression (anxiolytics and antidepressants), and administered the following questionnaires: Spanish adaptation [13] of the Charlson Combined Comorbidity Index [14], International Physical Activity Questionnaire (IPAQ), on preadmission activity [15] and the Pardo questionnaire [16] on pre-admission dietary and physical exercise habits. 

At hospital discharge, another specifically trained nurse recorded the days of hospital stay and administered the validated Spanish adaptation [17] of the EQ-5D-5L quality of life questionnaire [18], including the visual analog scale (VAS).

### 2.3. Measurement Instruments 

Combined Charlson Comorbidity Index [13] is a combined age and comorbidity index that considers 19 diseases (weighted from 1 to 6) and age (1 point per decade above 40 years of age). 

The short version of the IPAQ [15], designed for individuals aged between 15 and 69 years, contains four generic items and evaluates their activity level as low, moderate, or high (in MET units). Low activity = no or mild physical activity; moderate activity = ≥3 days of vigorous physical activity of ≥20 min/day or ≥5 days of moderate physical activity and/or walking for ≥30 min/day or ≥5 days of any combination of activity and walking (total of ≥600 MET-min/week); and high activity = activity of vigorous intensity for ≥3 days (≥1500 MET-min/week), or ≥7 days of any combination of walking and activities of moderate or vigorous intensity (≥3000 MET-min/week).

Pardo questionnaire [16] on life habits related to overweight and obesity contains 22 items grouped in the following five factors: (1) eight items on the calorie content of the diet; (2) three items on regular physical exercise; (3) six items on healthy eating, (4) two items on alcohol consumption (AC), and (5) three items on food consumption for psychological wellbeing (“emotional eating”). A five-point Liker scale (1–5) was used to respond to each item, adapting scores so that a higher result always indicated a healthier habit. 

EQ-5D-5L [17], which measures self-perceived health on the day of its administration, comprises two parts: a descriptive with five dimensions (mobility, personal care, daily activities, pain/discomfort, and anxiety/depression) with responses from 1 to 5 (higher score = worse status); and a VAS (from 0 = worst imaginable health status to 100 = best imaginable health status). 

The study complied with EU regulations (2016/679) and Spanish legislation (3/2018) on personal data protection and digital rights and was conducted in accordance with the 2013 revision of the Declaration of Helsinki (https://www.wma.net/what-we-do/medical-ethics/declaration-of-helsinki/; accessed on 13 July 2017). All subjects gave their informed consent to participate in the study, which was approved by the clinical research ethics committees of Andalusia and the four participating hospitals. 

## 3. Statistical Analysis

In a descriptive analysis, qualitative variables were expressed as frequencies and percentages and quantitative variables as medians with interquartile range (IQR [P25–P75]), applying the Kolmogorov-Smirnov test to check the normality of variable distributions. In bivariate analyses, Pearson’s chi-square test and Fisher’s test were used for qualitative variables and the Mann-Whitney test for quantitative variables. SPSS v.19 (IBM Corp., Armonk, NY, USA) was used for the data analyses, considering *p* < 0.05 as significant.

## 4. Results 

The study included 275 patients aged between 19 and 95 years, with a median age of 71 years (IQR [56–81]); 35.6% were with overweight (58.2% males) and 64.4% with obesity (52% males) at hospital admission. There was no statistically significant difference in sex distribution between the overweight and obesity groups.

It is estimated that around 1000 patients with overweight and obesity and no diabetes are annually admitted to the participating Intensive Medicine Departments. Therefore, the study population of 275 patients enrolled during the study period represents 13.75% (275/2.000) of the total number of patients admitted with overweight and obesity.

Table 1 exhibits the results of the descriptive and bivariate analyses. The groups did not significantly differ in: sociodemographic variables; consumption of medication for depression, 11.2% (*n* = 11) vs. 10.7% (*n* = 19) in patients with obesity, or anxiety 6.1% (*n* = 6) vs. 13% (*n* = 23) in patients with obesity, although the difference in anxiety medication was close to significant (*p* = 0.082); low physical activity by IPAQ, 63.3% (*n* = 62) vs. 69.5% (*n* = 123) in patients with obesity; median days of hospital stay (8 in each group); or median Charlson Comorbidity Index score (3 in each group). 

Pardo questionnaire results revealed healthier behavior in patients with overweight versus obesity in regular physical exercise (median of 1.66 vs. 1, respectively, *p* = 0.007), healthy eating habits (median of 3.67 vs. 3.5, *p* = 0.004), and emotional eating (median of 4.33 vs. 4, *p* = 0.014). There was no between-group difference in alcohol consumption (median of 3 in each group) (*p* = 0.113) (Table 1). 

Both groups obtained a median EQ-5D-5L score of 60 (Table 2), with a mean VAS value of 57.18. The percentage of patients with overweight versus obesity reporting category 1 (best situation) for each questionnaire dimension was 43.9% (*n* = 43) vs. 38.4% (*n* = 68) for mobility, 63.3% (*n* = 62) vs. 53.7% (*n* = 95) for personal care, 54.1% (*n* = 53) vs. 48.6% (*n* = 86) for daily life activities, 44.9% (*n* = 44) vs. 37.9% (*n* = 67) for pain or discomfort, and 53.1% (*n* = 52) vs. 48.6% (*n* = 86) for anxiety and depression. None of these differences reached statistical significance.

## 5. Discussion

In this study of patients admitted to an Intensive Medicine Department, weight-related habits before their admission were superior in those with overweight than in those with obesity.

In comparison to the patients with obesity, the Pardo questionnaire results showed that patients with obesity were less compliant with the basic features of a healthy diet. The WHO has published recommendations on the components of a healthy diet [19], although the relationship of certain food groups with overweight and obesity continues to be under debate [20,21,22,23]. The groups did not differ in their alcohol consumption, which was found to be higher in people with obesity versus overweight in a study in the USA [24]. This discrepancy may be attributable to differences in the type of patient and their comorbidities and/or in health care recommendations.

Emotional eating was more frequent in the present patients with obesity than in those with overweight. The association between emotions and food is well documented [25,26], and binge eating disorder [27] and negative states of mind have been associated with obesity [28]. Clinicians should pay special attention to the mental state of people with excess weight, given the bidirectional risk between obesity and depression, which is higher in females [6]. However, no statistically significant differences were observed between groups in the “anxiety/depression” dimension of the EQ-5D-5L, although this questionnaire does not discriminate between the two disorders. Patients with anxiety and depression were receiving treatment that had been prescribed by their primary care physician. European guidelines for the management of obesity in adults published in 2015 [29] recommends the specialist treatment of those with anxiety, depression, and/or stress in a life-long multidisciplinary approach. 

The patients with overweight and obesity did not significantly differ in any dimension of the EQ-5D-5L HRQoL questionnaire at discharge, unlike in other studies [5,30,31], which may be explained by differences among study populations. However, it should be kept in mind that people with obesity are more prone to suffer pain [5] and have poorer mobility [32], with an increase in bidirectional risk over time between weight and mobility disability [33]. A high percentage of both groups in the present study reported pain and suffered from mild, moderate, or severe mobility impairment, which should be taken into account in their post-discharge care. 

With regard to the self-evaluation of their health status, the present patients had a mean VAS of 57.18, around 18–19 points worse than the mean values reported for the Spanish general population in the 2011–2012 National Health Survey [34], which were 76.16 for Andalusia and 75.83 for Ceuta.

Patients with overweight practiced more exercise in comparison to those with obesity, in agreement with previous studies [22]. However, there was no between-group difference in IPAQ score, which may be explained by the large percentage of patients aged over 70 years, given that this questionnaire is more appropriate for individuals below this age. The practice of physical activity several times a week has been related to weight loss [21,35].

No between-group difference was found in sociodemographic characteristics, consumption of depression medication before admission, length of hospital stay, or Charlson Comorbidity Index score.

## 6. Study Limitations 

The recruitment period was extended from one to two years due to difficulties in obtaining an adequate sample of patients who met eligibility criteria, especially the absence of diabetes and an adequate cognitive status for questionnaire completion. The borderline results obtained for some variables might have reached statistical significance with a larger sample size. A further potential limitation is that the data were gathered by different nurses, although they all received specific training from a single researcher (CHE) to minimize variations.

## 7. Conclusions

Before their hospital admission, patients with obesity were less compliant with the basic rules of healthy eating, engaged in more emotional eating, and practiced less physical exercise in comparison to those with overweight. There was also a close-to-significant tendency for a higher frequency of anti-anxiety medication in patients with obesity versus overweight. Greater efforts are warranted to prevent an increase in the BMI of patients, paying special attention to their state of mind.

## Figures and Tables

**Table 1:** Descriptive variables considered and bivariate analysis between patients with overweight and obesity.

Variables	Overweight (BMI = 25–29.9)	Obesity (BMI ≥ 30)	*p*	Effect Size
	*n* = 98 (35.6%)	*n* = 177 (64.4%)		
Male sex	57 (58.2%)	92 (52.0%)	0.324	0.12
Schooling				
No schooling	24 (24.5%)	58 (32.8%)	0.096	0.27
Primary schooling	54 (55.1%)	74 (41.8%)		
Secondary schooling/university education	19 (19.4%)	44 (24.9%)		
Missing	1 (1%)	1 (0.6%)		
Work Situation				
Unemployed	11 (11.2%)	15 (8.5%)	0.665	0.11
Professionally active/Student	16 (16.3%)	34 (19.2%)		
Retired/Permanent disability	68 (69.4%)	128 (72.3%)		
Missing	3 (3.1)	0 (0%)		
Cohabitation				
Alone	13 (13.3%)	21 (11.9%)	0.714	0.04
Accompanied	83 (84.7%)	154 (87%)		
Missing	2 (2%)	2 (1.1)		
Depression medication-Yes	11 (11.2%)	19 (10.7%)	0.88	0.02
Anxiety medication-Yes	6 (6.1%)	23 (13%)	0.082	0.29
IPAQ Physical Activity before Hospital Admission				
Low activity	62 (63.3%)	123 (69.5%)	0.303	0.21
Moderate activity	20 (20.4%)	39 (22%)		
High activity	11 (11.2%)	11 (6.2%)		
Missing	5 (5.1%)	4 (2.3%)		
Median [P25–P75]				
Age	72 [59–83]	69 [55–80]	0.105	0.1
Days of hospital stay	8 [5–10]	8 [6–10.75]	0.558	0.04
Charlson Comorbidity Index	3 [1–5]	3 [1–4]	0.798	0.02
Pardo Questionnaire of Habits Related to Obesity and Overweight before Hospital Admission				
Calorie content of diet	2 [1.75–2.66]	2 [1.75–2.44]	0.301	0.06
Regular/systematic practice of physical exercise	1.66 [1–2.33]	1 [1,2]	0.007	0.16
Compliance with basic rules of healthy eating	3.67 [3.25–4.17]	3.5 [3.17–3.83]	0.004	0.18
Alcohol consumption	3 [3–4]	3 [3–3.87]	0.113	0.09
Food intake for psychological wellbeing	4.33 [4–5]	4 [3–4.67]	0.014	0.15

Me (P25–P75): Me = Median [interquartile range]; Note: non-responders (missing data) were not considered in the chi-square test calculations.

**Table 2:** EQ-5D-5L L dimensions for self-perceived health between patients with overweight and obesity at discharge.

Response Options	Overweight *n* (%)	Obesity *n* (%)	*p*	Effect Size
	98 (35.6)	177 (64.4)		
Mobility			0.401	0.24
No problems walking	43 (43.9)	68 (38.4)		
Mild problems walking	21 (21.4)	33 (18.6)		
Moderate problems walking	14 (14.3)	40 (22.6)		
Severe problems walking/cannot walk	20 (20.4)	35 (19.8)		
Missing	0 (0)	1 (0.6)		
Personal Care			0.296	0.27
No problems washing or getting dressed	62 (63.3)	95 (53.7)		
Mild problems washing or getting dressed	10 (10.2)	27 (15.3)		
Moderate problems washing or getting dressed	10 (10.2)	28 (15.8)		
Severe problems/inability to wash or dress themselves	16 (16.3)	27 (15.3)		
Daily Activities			0.847	0.12
No problems carrying out daily activities	53 (54.1)	86 (48.6)		
Mild problems carrying out daily activities	15 (15.3)	28 (15.8)		
Moderate problems carrying out daily activities	11 (11.2)	25 (14.1)		
Severe problems/inability to carry out daily activities	19 (19.4)	36 (20.3)		
Missing	0 (0)	2 (1.1)		
Pain/Discomfort			0.21	0.27
No pain or discomfort	44 (44.9)	67 (37.9)		
Mild pain or discomfort	33 (33.7)	54 (30.5)		
Moderate pain or discomfort	16 (16.3)	39 (22)		
Strong/extreme pain or discomfort	4 (4.1)	17 (9.6)		
Missing	1 (1)	0 (0)		
Anxiety/Depression			0.495	0.2
Not anxious or depressed	52 (53.1)	86 (48.6)		
Mildly anxious or depressed	27 (27.6)	61 (34.5)		
Moderately anxious or depressed	13 (13.3)	16 (9)		
Very anxious or depressed	6 (6.1)	13 (7.3)		
Missing	0 (0)	1 (0.6)		
EQ-5D-5L VAS Median [P25–P75]	60 [50–70]	60 [45–70]	0.831	

## Data Availability

The underlying data are available on request.
